# Knowledge and dispensing practice of community pharmacists towards antipsychotic medicines in a Nigerian metropolitan city – a cross-sectional study

**DOI:** 10.1186/s12913-023-10480-0

**Published:** 2023-12-21

**Authors:** Joseph Olasupo, Faith Taiwo, Akinniyi Aje, Titilayo O. Fakeye

**Affiliations:** Department of Clinical Pharmacy and Pharmacy Administration, Faculty of Pharmacy, University of Ibadan, Ibadan, Nigeria

**Keywords:** Antipsychotics, Community pharmacies, Over-the-counter dispensing, Prescription-only-medications, Regulation

## Abstract

**Background:**

Antipsychotic drugs are prescription-only-medications which require valid prescriptions before it can be obtained from a pharmacy. On the other hand, community pharmacists in developing countries have sometimes been implicated in over-the-counter dispensing of prescription-only-medications.

**Objective:**

This study investigated the accessibility of antipsychotic drugs without prescriptions from community pharmacies, and the factors responsible for the over-the-counter dispensing of antipsychotics by community pharmacists.

**Methods:**

An exploratory cross-sectional mixed method survey design using pretested structured questionnaires among 119 community pharmacists, simulated patients in 119 community pharmacies, and one-on-one in-depth interview among eleven (11) community pharmacist-owners/superintendent pharmacists were utilized for data collection. The knowledge of the pharmacists on antipsychotics including classification, side effects, and dispensing practices were explored. Qualitative data was analyzed with thematic analysis, while quantitative data was analyzed using descriptive statistics.

**Results:**

Majority of the community pharmacists (87.4%) showed good knowledge of antipsychotics as it relates to the different classes and the side effects peculiar to each class. Antipsychotic medications were dispensed by 85 (71.4%) of community pharmacists without a prescription. One-on-one in-depth interview sessions with community pharmacist owners/superintendent pharmacists demonstrated that community pharmacists are knowledgeable about antipsychotics and their side effects. Reasons given for dispensing this class of drugs without prescription included emergencies, and knowledge of the person as being on the drugs long-term. About 4% pharmacists were adamant on dispensing only with prescription.

**Conclusion:**

Community pharmacists in Ibadan metropolis readily dispense antipsychotics without valid prescriptions despite having an optimal knowledge about the negative implications of doing so. This could be due to weak legislation and regulation of drug laws. There is a need for more stringent regulations as well as adequate sensitization about the negative effects of inappropriate dispensing of prescription-only-medications.

**Supplementary Information:**

The online version contains supplementary material available at 10.1186/s12913-023-10480-0.

## Background

Antipsychotics, also known as neuroleptics, are a class of medications primarily used to manage psychosis and its associated symptoms such as delusions, hallucinations, paranoia or disordered thought [1]. They are primarily recommended as the first line drug used in treating schizophrenia. However, they are also prescribed off-label (mostly due to their calming and mood-balancing effect) for a broad range of indications including bipolar disorder, depression, obsessive-compulsive disorders, post-traumatic stress disorder, severe agitation and psychotic experience in dementia [2]. They principally act by blocking D2 receptors in the dopaminergic as well as in the serotonergic pathways of the brain [3]. With respect to their mechanism of action, side-effects and time of discovery, antipsychotics can be broadly classified into atypical antipsychotics (e.g. olanzapine, risperidone, clozapine, quetiapine etc.) and typical antipsychotics (e.g. chlorpromazine, haloperidol, loxapine) [4]. Antipsychotics, which regulate neurotransmitters and stimulate receptors in the central nervous system, are associated with serious side effects [5, 6]. Patients on antipsychotics may develop extrapyramidal side effects that may be permanent and difficult to reverse in some cases [7–9]. They can also complicate or initiate metabolic syndrome in certain conditions, worsening pre-existing conditions [10–13]. As drug custodians, community pharmacists should have a comprehensive knowledge of all classes of drugs, their side effects, and the precautions to be taken when dispensing them. The most common side-effects associated with antipsychotics are tremors, weight gain, sedation, impaired sexual function, dizziness and tardive dyskinesia. Atypical or second-generation antipsychotics are associated with milder side effects, except in the case of weight gain [14]. Although they do not provide the euphoria effect that addictive drugs do, they are associated with considerable withdrawal effects making it difficult to be discontinued abruptly [15]. Although antipsychotics are not considered to be addictive, studies have shown their tendencies to be abused and misused due to their sedating and tranquilizing effects [16, 17].

Inappropriate use of antipsychotics carries the potential for fatal adverse effects, withdrawal symptoms and sudden death due to drug-induced ventricular arrhythmia [18].

In many parts of the world including Nigeria, antipsychotics are classified as prescription-only-medications (POMs). Also, community pharmacists are not expected to dispense prescription-only medications without valid prescriptions. They are considered drug experts and work to ensure patients receive the best therapeutic plan prepared by a physician in collaboration with other healthcare professionals. When dealing with more serious ailments, such as psychosis and psychotic-related conditions, community pharmacists should refer patients to hospitals for better care by qualified healthcare professionals. Psychosis is a complex condition managed by a specialist, and general practitioners are unable to handle it due to the complex nature of the disease and the elusive parameters required for accurate diagnosis [19–21].

Regulatory control over sale of medications is poor in most developing countries [22, 23] highlighting the need to examine how accessible antipsychotics are to the public as over-the-counter drug classified as prescription-only medications in Nigeria. This study evaluated access to antipsychotic medications without prescriptions in community pharmacies in Ibadan metropolis located southwestern Nigeria, using qualitative and quantitative methodologies. The level of knowledge of the community pharmacists, superintendent pharmacists/pharmacist owners on use and the right dispensing practices of antipsychotics among others, were all evaluated.

## Method

### Ethical approval

Ethical approval numbers UI/EC/21/205 and UI/EC/21/0212 were obtained from the University of Ibadan/University College Hospital Institutional Review Board.

### Study design

This was an exploratory study involving three methods of data collection: one-on-one in-depth interview, structured questionnaire and the simulated patient (observational) techniques. Simulated patients were used in order to remove the Hawthorne effect. The pharmacies for the observational method and questionnaire were selected using clustered sampling techniques.

### Study setting

The study took place in Ibadan, a metropolitan city and the third-largest city in Nigeria with a total population of 3,649,000. There are eleven (11) local government areas in Ibadan. They are Ibadan North, Ibadan North-East, Ibadan North-West, Ibadan South-East, Ibadan South-West, Akinyele, Egbeda, Ido, Lagelu, Ona-Ara and Oluyole local government areas.

The study covered all the eleven local governments. Community pharmacists in these eleven local governments were selected for the study.

### Study population

The study population were registered community pharmacists who were licensed to practice. Pharmacist interns, corp pharmacists, student pharmacist on industrial training were all exempted from the study.

### Sample size determination

Using the list of registered and licensed community pharmacists practicing within the eleven local government in Ibadan metropolis in 2019 obtained from the Pharmacy Council of Nigeria, 151 retail community pharmacies were fully registered and licensed. The sample size for the pharmacies to be visited by simulated patient and the community pharmacists to whom structured questionnaire were administered was obtained by using the Raosoft Sample Size Calculator® (www.raosoft.com). Using a margin of error of 5%, confidence level of 95%, and response distribution of 50%, and an allowance of 10% nonresponse led to a sample size of 120 pharmacies/community pharmacists. One community pharmacist owner/superintendent pharmacists per local government was selected for the interview giving a total of eleven interviewees.

### Research instruments

For the in-depth interview involving the pharmacist-owners/superintendent pharmacists, open-ended questions addressed the reasons for dispensing antipsychotic drugs both as prescription or over-the-counter medications, and their knowledge and attitude towards dispensing as such.

The questionnaire was retested among 12 community pharmacies. The pharmacists involved in the pretest were excluded from the study. Three questions under attitude were rephrased to remove ambiguity. Also, content validity of the questionnaire and interview questions was carried out by six faculty members of the Department of Clinical Pharmacy and Pharmacy Administration, Faculty of Pharmacy, University of Ibadan, Nigeria. The resulting structured questionnaire (Appendix A) were self-administered among the community pharmacists (not more than one pharmacist per pharmacy, and only one pharmacy per pharmacies with more than one branch). The questionnaire, which investigated the pharmacists’ knowledge on antipsychotics used multiple choice questions. Section A addressed the demographics of the pharmacists, section B investigated the pharmacists’ knowledge on antipsychotics using multiple choice questions and section C assessed the pharmacist’s reasons and rationale for dispensing antipsychotics as either over-the-counter drugs or prescription-only medicines. The section C consisted of statements with “yes” or “no” responses. Seventeen questions were administered to evaluate the pharmacists’ knowledge on antipsychotics.

For the observational technique, each simulated patient went to the assigned community pharmacies, requested for antipsychotics without prescription using different approaches.

### Data collection

The study spanned from April 2022 till September 2022. The community pharmacies were selected using clustered sampling methods for the observational (simulated patient) and structured questionnaires in the eleven local governments. Briefly, the pharmacies in each local government were determined from the list of registered and licensed pharmacies. Proportional method was used to determine the number of pharmacies that was visited in each local government. The visited pharmacies were selected using purposive sampling.

Five final year pharmacy students that have been well trained to perform the role of simulated patients were used to document whether or not antipsychotic agents will be sold over-the-counter or the pharmacist will request for a prescription. Four common antipsychotics (two each of typical and atypical) in Nigeria namely olanzapine, risperidone, haloperidol, chlorpromazine were selected as the choice of drugs for the simulated patients. Responses to requests by simulated patients such as “My refill from hospital has finished. Can I get a pack of **drug A** (mentioning one of the names of the four selected antipsychotics) as I am out of my drugs?” or “Doctor wrote me a prescription for **drug A** (mentioning one of the names of the four selected antipsychotics) but I have misplaced it somewhere. I remember the name of the drug and the dose. I want to buy 2 packs pending the time I will go see my doctor again” or “Please sell me just 2 sachets, my uncle called me to get it for him on my way”. The responses of the pharmacist were categorized as either “pharmacist sold the drug”, “pharmacist refused to sell without prescription” or “pharmacist sold the drug after much persuasion without prescription” or “drug is out of stock”. If the community pharmacist demands a prescription, the community pharmacist was further persuaded to dispense without prescription. The responses were recorded.

After having been visited by a simulated patient, the questionnaire was taken to the pharmacy the next day by a different research assistant, and if the pharmacist was not available, the research assistant goes back the next day. Questionnaires were not dropped if the pharmacist could not be reached. The research assistant waited for the questionnaire to be filled if the pharmacist were met at the premises. The filled questionnaires were collected immediately.

For the interview, one community pharmacy per local government was randomly selected by ballot. The pharmacist-owner or the superintendent pharmacist of the eleven pharmacies were contacted and interviewed by appointments. The reason for the interview were not disclosed until the interview started. The interview took place in the consultation room/pharmacist office. The responses provided by the community pharmacists was recorded using a recording application on a smartphone after permission was obtained. The data collected was transcribed and converted to a written form, which was then further analyzed.

All participants i.e. the community pharmacists (who filled the questionnaires) and the superintendent pharmacists/pharmacist-owners who were interviewed gave their informed consent.

### Data analysis

The data obtained from the structured questionnaire was coded and entered into Statistical Package for Social Sciences (SPSS) software, version 20. Descriptive statistics was utilized to describe the performance of the community pharmacists based on the scores obtained from the questions on knowledge. Each correct answer was scored “1”, while an incorrect answer was scored “0” for the 17 questions. These scores were converted to percentages and the range of scores for evaluation was as follows; (0–49.9% - poor; 50–69.9% - fair; 70–89.9% - good; 90–100% -excellent). Frequency counts, percentages, mean and standard deviation and variance of the scores gotten from the responses were determined. One-way Analysis of Variance (ANOVA) was used to evaluate the effects of the demographic variables on the attitude and scores. The level of significance was at *p* < 0.05.

Descriptive analysis was used to evaluate the occurrence of over-the-counter dispensing antipsychotic drugs, as well as the choice of dispensing when persuaded by the simulated patient. Frequency and percent frequency were used to summarize the data.

The output from the interviews was analyzed using thematic analysis. An experienced transcriber was employed who produced the written copies of the audio generated from the in-depth interview. The output from the interviews was analyzed using thematic analysis. The coding was initiated by getting familiarized with the data collected and transcribed from the interview. Preliminary codes were assigned to the data in order to describe the content. Similar codes were generated and brought together. The similar codes eventually were used to form themes which were reviewed, defined, and named. The representative responses of the pharmacists for each theme was documented with each participant (P) assigned number 1 to 11.

The data obtained from the questionnaire and the simulated patients were compared in order to get a true picture of the attitude and exact practice of dispensing antipsychotic drugs by the community pharmacists.

## Results

At the end of the study, 122 questionnaires were filled. Only 119 could be analyzed giving a response rate of 97.5%. Three questionnaires were discarded due to the non-filling of all the questions on knowledge. Three simulated patients data from the same pharmacies were also discarded.

### Demographics

There were 52.9% (63) males and 47.1% (56) females. Majority of the pharmacists 80 (67.2%) had community pharmacy experience ranging from 1 to 5 years. Also, 95 (79.2%) of the community pharmacists had no other tertiary qualification aside B.Pharm. The other demographic variables are shown in Table 1.

**Table 1:** Demographic information of participants

Demographic factor	Variable	Frequency (Percentage)
Gender	Male	63 (52.9)
	Female	56 (47.1)
Community Pharmacy Experience	1–5 years	80 (67.2)
	6–10 years	23 (19.2)
	> 10 years	16 (13.3)
Educational Qualifications	B.Pharm only	95 (79.8)
	B.Pharm and other qualifications	24 (20.2)

### Knowledge and attitude

On the knowledge base, more than half of the pharmacists were not familiar with the third generation antipsychotic agent, aripiprazole, the adjunct medications given with antipsychotics when there is aggression, or the generation of antipsychotics with more neurological side effects. Their performance on knowledge of antipsychotics is shown in Tables 2 and 3. Table 3 shows the scores obtained based on their demographics with 104 (87.4%) scoring ≥50%. The pharmacists’ responses to attitude to dispensing antipsychotics is shown in Table 4. Almost all, 109 (92.4%), the pharmacists would dispense generic/cheaper antipsychotics over-the-counter while the originator’s brands or “expensive” generics such as risperidone, olanzapine would be sold only with prescriptions. Also, about 73% would dispense OTC if they believe that the patients know what they want, if the patient “begs” (71.4%), when it is parents buying for their children (75.6%) and if the patient “appears knowledgeable” (73.1%). Some pharmacists would go as far as switching classes of antipsychotics if the patient complains about the prescribed one without referring the patient to the prescriber. Table 4 shows the grade obtained by the pharmacists on the knowledge on antipsychotics. The grades are shown based on gender, educational qualification and experience as community pharmacists.

**Table 2:** Knowledge of the pharmacists on various questions on antipsychotics

Question Statement	Correct Response Frequency (% frequency)
The following are pairs of antipsychotics (Haloperidol and Chlorpromazine)	71 (59.7)
Benzodiazepines are classical examples of antipsychotics	72 (60.5)
The following best describes a major side effect associated with second generation antipsychotics	81 (68.1)
Second generation antipsychotics are marked majorly with the following adverse effects	58 (48.7)
Aripiprazole is an example of third generation antipsychotic	23 (19.3)
Mood disorders e.g. depression should better be treated with tricyclic antidepressants	70 (58.8)
Schizophrenia is better treated with antipsychotics	96 (80.7)
Antipsychotics are used in the treatment of schizophrenia	108 (90.8)
The following except one should not be done for someone just showing signs of psychosis	64 (53.8)
Antipsychotics can be sold as over-the-counter drugs (OTCs).	96 (80.6)
Antipsychotics should be stopped once the signs and symptoms start to cease.	107 (89.9)
Atypical antipsychotics are more associated with neurologic side effects then typical antipsychotics.	58 (48.7)
It is safer to place a patient with diabetes mellitus on atypical antipsychotics than on typical antipsychotics.	62 (52.7)
Typical antipsychotics are marked with less neurologic effects.	103 (86.6)
Butyrophenones e.g. Haloperidol are classical examples of atypical antipsychotics.	84 (70.6)
Sometimes, as a community pharmacist, it is better to initiate antipsychotic therapy in a patient especially when the signs are recurring.	87 (73.1)
It is advisable to give anxiolytics e.g. benzodiazepines alongside with antipsychotic medications especially in patients that show physical aggression.	18 (15.1)

**Table 3:** Scores obtained by the pharmacists on knowledge of antipsychotics

Variable		Rank Frequency (% frequency)			
		Excellent	Good	Fair	Poor
Gender *n* = 116	Female	15 (12.9)	29 (25.0)	6 (5.2)	3 (2.6)
	Male	14 (12.1)	23 (19.8)	17 (14.7)	9 (7.8)
Educational Qualifications *n* = 117	B.Pharm Only	20 (17.1)	47 (40.2)	18 (15.4)	10 (8.5)
	Additional Qualifications	9 (7.7)	6 (5.3)	5 (4.3)	2 (1.7)
Years of Experience *N* = 117	1–5 years	15 (12.8)	42 (35.9)	15 (12.8)	8 (6.8)
	6–10 years	8 (6.8)	8 (6.8)	6 (5.1)	1 (0.9)
	> 10 years	6 (5.1)	3 (2.6)	2 (1.7)	3 (2.6)

**Table 4:** Attitude of participants in dispensing antipsychotics

Statements	No Frequency (%)	Yes Frequency (%)
Sometimes, I dispense antipsychotics as over-the-counter drugs, especially in an emergency situation. For instance, if a known antipsychotic patient ran out of his or her medication and is in urgent need of it.	91 (76.5)	28 (23.5)
I do not request for prescription for antipsychotics especially for known patients.	49 (41.2)	70 (58.8)
I believe every patient that walks into the Pharmacy requesting for antipsychotics know what they want to use it for, so I dispense it when they request for it.	32 (26.9)	87 (73.1)
I dispense the cheaper classes of antipsychotics (Chlorpromazine, Haloperidol) as over-the-counter drugs but when it comes to the purchase of the expensive ones (Risperidone, Olanzapine), I ask for prescription.	9 (7.6)	110 (92.4)
I readily dispense antipsychotics especially then the patient begs that he or she is in dire need of it.	34 (28.6)	85 (71.4)
Sometimes, parents come to get antipsychotics for their children, and I readily dispense it especially when they tell me it’s for their children.	29 (24.4)	90 (75.6)
I readily dispense antipsychotics especially when clients give good reports about the last brand they bought.	16 (13.4)	103 (86.6)
I readily dispense antipsychotics to patients when they tell me they’ve been on it for long.	50 (42.0)	69 (58.0)
I can switch to another class of antipsychotics when a patient gives complaints about the one he/she currently on.	21 (17.6)	98 (82.4)
I will rather dispense chlorpromazine to an obese patient rather than dispense clozapine because of the weight gain associated with clozapine.	26 (21.8)	93 (78.2)

### Simulated patient outcome

Thirty-one (26.1%) community pharmacies did not have stock of the requested drugs. Out of the thirty-one community pharmacies that did not have stock of the antipsychotic drugs requested, twenty-one (67.7%) indicated willingness to sell without prescription if it were on stock. Two other community pharmacists asked for prescription despite not having stock; indicating that they might have had no intention to sell even if they were in stock. Out of the ninety-one community pharmacies that had stock of the antipsychotic demanded, eighty-five (93.4%) were ready to dispense without prescription. Six community pharmacies out of the 91 community pharmacies that had stock of antipsychotics demanded that a prescription is shown before any antipsychotic drug sale can be honored. Out of the six community pharmacies that asked for a valid prescription, three (50%) dispensed after further persuasion by the simulated patient, while the other three (50%) insisted that a sale cannot be made unless a valid prescription is presented. This gave a total of five pharmacies who would sell only on presentation of a valid prescription.

### In-depth interview

The responses of the eleven community pharmacist owners/superintendent pharmacists vis-à-vis dispensing of antipsychotics as prescription-medications only was as varied as the responses to the questionnaire by the community pharmacists. The major themes obtained are “Referral and Discretion”, “License limits and Specialization” and “Adherence” (Table 5). The themes are discussed below:

#### License limits and specialization

“*Antipsychotics should be prescribed before it can be dispensed”*(P1, P9) and “*pharmacists are not the right persons to recommend antipsychotics*”(P11). Among the community pharmacists that were against over-the-counter dispensing of antipsychotics in the interview sessions, statements such as “*it is important that community pharmacists assume responsibility for any drug that goes out of the pharmacy*” (P1) placing strong emphasis on the reason why “*community pharmacists must be mindful and professional at every point of their service to the public*” (P9). The most common reason for not recommending antipsychotic drugs that was highlighted by the participants 1, 9 and 11 was that “*any business relating to antipsychotics must only be handled by specialists and not pharmacists*”. They also explained how “*complex psychotic-related disorders are”* (P9), “*…. it is only specialists (*psychiatrists*) who can determine which ones are right for the condition”* (P9, P11) and that “*antipsychotics should not be recommended by pharmacists at all”* (P1, P9, P11).

#### Referral and discretion

P1, P7, and P8 were of the opinion that “*pharmacists can use their discretion to decide when to dispense and when not dispense antipsychotics”*. They also believed that “*community pharmacists can dispense on compassionate grounds as long as the pharmacists can use discretion to know if the patient needs it or not”* (P7, P8) and that “*sometimes, one may not be able to wait for prescriptions* (if there are) *…. emergency*”(P1). P3, P6 and P10 believed that “*pharmacist can recommend antipsychotic drugs as long as proper monitoring* (P6), *documentation* (P3) *……. evaluation of the patient is done*”(P10). P4 believes that “*balance can be met in dispensing antipsychotic drug as prescription or over-the-counter drugs*” and (P5) “*in cases of emergencies* (acute), *first doses may be dispensed*” and *“….patients may be given 24-hour doses to tide him over till the next day”*(P1, P4, P8).

#### Adherence

Participants 3 and 4 explained that the “*cost to see physicians is very high in Nigeria”* (P3) and “*it is unfair if patients are denied the choice to use their medications when needed*” (P3, P4). P2, P3, P4 and P5 summarized that “*failure to dispense to such patients* (who do not have prescriptions) *is an act of encouraging non-adherence*”.

**Table 5:** Themes obtained from one-on-one in-depth interview

Theme	Codes
**Referral and Discretion**	1. Referral 2. Antipsychotics specialist business, not pharmacists. 3. Dispense without prescription 4. Recommend antipsychotics 5. ‘First aid’ in the management of psychotic related signs 6. Breach of trust 7. Antipsychotics as off-label
**License limits and Specialization**	1. Negative implications of over-the-counter dispensing of antipsychotics 2. Specialization 3. Need for knowledge base for community pharmacist
**Adherence**	1. Encouraging non-adherence 2. Cost of seeing physician 3. Unfairness to patient

## Discussion

For many mental disorders such as depression, bipolar disorder and schizophrenia, medicines remain the major modality of treatment. It therefore follows that community pharmacists contribute to the management of mental disorders, hence the need to dispense antipsychotics only against a valid prescription, while offering counsel. Previous literature reviews of community pharmacy settings have highlighted this important clinical role of pharmacists [16, 17].

A study carried out by Mekonnen et al. [24] in Ethiopia evaluated the knowledge of community pharmacists on antipsychotics and psychotropic agents. The pharmacists had good knowledge of antipsychotics and side effects peculiar to each class. This was similar to the results obtained by Mooney et al. [25] where majority of the community pharmacists in Texas, America had a good knowledge on the use of antipsychotics but in contrast with the results obtained by Mekonnen et al. [24], 52.1% had poor knowledge. Other studies carried out by Akinyandenu et al. [22], Dameh et al. [26] and Levine [27] evaluated the accessibility of prescription-only medicines over-the-counter from community pharmacies. Most of the pharmacists in the present study understand the side effects associated with typical and atypical antipsychotics but were unaware of the third-generation antipsychotics (e.g. Aripiprazole).

Switching patients to another class of antipsychotics when there are complaints about the prescribed drug(s) is definitely not one of the roles of the pharmacist, especially without the prescriber’s knowledge. The harmful potentials of dispensing of antipsychotics without prescription are not far-fetched. They include the potential for drug abuse, drug misuse, drug dependence with considerable economic consequences, some of which were mentioned in the one-on-one in-depth interview of community pharmacist owners/superintendent pharmacists. Also, antipsychotic drugs are associated with fatal adverse effects if improperly used and may lead to a general increase in disability-adjusted life years and preventable deaths [9]. Antipsychotics and other related drugs have also been implicated in recreational uses and addiction [16, 17, 28, 29] which can be further complicated by unencumbered over-the-counter access.

The simulated patient method was an observational technique in which the community pharmacists were not aware that the “client” is actually a simulated patient. This ensured that the community pharmacist responded without any bias. The simulated patient method confirmed the results from the structured questionnaire which showed that the majority of the 119 community pharmacists were willing to dispense antipsychotics without a prescription. Despite all the reasons communicated by the community pharmacists owners/superintendent pharmacists in the interview, antipsychotic drugs are not meant to be dispensed without prescription under any condition. While some of the community pharmacists supported that antipsychotic drugs should only be dispensed over-the-counter conditionally, some were not in agreement. This is inconsistent with the findings from the simulated patient method since the pharmacists were not aware that they were being assessed by the “client”.

The number of OTC dispensing of antipsychotic is very high and could be taken as a reflection of the community pharmacy practice. This behavior is consistent with studies that assessed the over-the-counter dispensing of other classes of drugs in Nigeria, as well as in other developing countries [30–34]. It also demonstrated a trend similar to a study conducted in Saudi Arabia which evaluated the sale of an antipsychotic (fluoxetine). It was discovered that the majority of the community pharmacists dispensed fluoxetine without prescription, buttressing the fact that over-the-counter dispensing of POMs, including antipsychotic drugs is very prevalent in developing countries. Majority of the community pharmacists are fully aware of the possible negative effects when antipsychotics are dispensed without prescription. The awareness that legal actions can be taken, the economic implications and wastage of resources, the high risk of abuse, misuse and overdose are among the major points made by the community pharmacist owners/superintendent pharmacists. This is consistent with the carefully identified risks that have been associated with the over-the-counter dispensing of antipsychotics in Saudi Arabia [35, 36].

The number of community pharmacists that asked for a prescription is unacceptably low. Asking for a prescription following the request was the right thing to do. While persuasion may be emotionally stressful to community pharmacists, it is one of the most common encounters that they will face in the course of practice. Hence, one of the key attributes community pharmacists must have is understanding how to manage pressure and inappropriate demands. Telling the requestor that the “drug is not available” was usually the method used by the community pharmacists to refuse dispensing prescription medicines which has been found effective in managing situations when pressure is being exerted to sell POMs over-the-counter [37] which may not be the right approach since the pharmacists owe the duty of explaining why some drugs will not be dispensed OTC. Only three out of ninety-one did not dispense and showed no plan to dispense without prescription. This number is of a major concern to the distribution of POMs in Nigeria. Despite all the sentiments expressed by the interviewed pharmacists, their responses were not in tandem with the actual dispensing behavior of majority of the 119 retail community pharmacists.

It can be established, based on these findings, that the majority of community pharmacists in Ibadan, Oyo state are willing to dispense antipsychotics without prescription. The interview sessions revealed that the eleven (11) community pharmacists have an acceptable level of knowledge about antipsychotic drugs and their side effects. This was also borne out by the responses obtained from the questionnaire. However, their attitude and behavior toward dispensing is of a major concern and contrary to the standards of professional pharmacy practice. The prevalent state of inappropriate over-the-counter sale/dispensing of antipsychotic drugs by community pharmacies is mainly due to the weak implementation of existing legislation and regulation of drug distribution within the country. The interviewees claimed that it is wrong to improperly dispense antipsychotics, indicating that the majority of community pharmacists know the law, what is the right and standard practice. Until the gap is adequately addressed and the over-the-counter dispensing of antipsychotic is curbed by adequate regulations and implementations, the public is at the risk of drug misuse, abuse of a very sensitive and potentially harmful class of drugs.

The interview method used in the study is open to Hawthorne effect. The use of interview, structured questionnaire and simulated patients gave a clear picture of the over-the-counter dispensing of an important class of prescription-only medicine by community pharmacists.

## Conclusion

Community pharmacists in Ibadan metropolis have a good and optimal level of knowledge about antipsychotics and the common side effects associated with them. The general attitude of community pharmacists in over-the-counter dispensing of antipsychotics, a class of prescription-only medicines, is very poor, with most community pharmacists willing to sell without prescriptions.

### Electronic supplementary material

Below is the link to the electronic supplementary material.

Supplementary Material 1

## Data Availability

The data are available on request. You may contact Titilayo Fakeye at titifakeye@gmail.com. All experiments were performed in accordance with relevant guidelines and regulations.

## Abbreviations

P: Participants
