# Supplementary material for: Knowledge and dispensing practice of community pharmacists towards antipsychotic medicines in a Nigerian metropolitan city – a cross-sectional study
Source: BMC Health Serv Res. 2023 Dec 21;23:1450. doi: 10.1186/s12913-023-10480-0 (PMC10740283; doi:10.1186/s12913-023-10480-0)
Supplement: Supplementary file 1 — Supplementary Material 1 [file 12913_2023_10480_MOESM1_ESM.docx]

**APPENDIX A**

**ASSESSMENT OF ACCESSIBILITY TO ANTIPSYCHOTIC DRUGS FROM COMMUNITY PHARMACY IN IBADAN, OYO STATE.**

I am presently carrying out a study on “Assessment of accessibility of antipsychotic drugs from community pharmacies in Ibadan using quantitative method.” I will be grateful if you could kindly fill the questionnaire in order to get information needed for the study. Please, note that any information obtained from you is for research purposes only and will be treated with utmost confidentiality.

Thank you.

**SECTION A: DEMOGRAPHICS**

Age: _______________

Gender: Male ( ) Female ( )

Years of experience as a Community Pharmacist: ____________ years

Additional Educational qualification(s) apart from B.Pharm: _____________

Year of graduation: ________________

**SECTION B: THIS SECTION WILL EVALUATE YOUR FAMILIARITY WITH ANTIPSYCHOTICS**

***Kindly underline the correct answer for questions 1-10 below, and tick “Yes” or “No” for questions 11-20.***

1. The following pairs are examples of first generation antipsychotics **except**
2. Haloperidol and Chlorpromazine.
3. Thioridazine and Clozapine.
4. Loxapine and Fluphenazine.
5. Risperidone and Olanzepine.
6. Benzodiazepines are classical examples of antipsychotics.
7. True
8. False
9. I don’t know.
10. The following pairs of antipsychotics are not atypical antipsychotics **except**
11. Molindone and Olanzepine
12. Aripiprazole and Clozapine
13. Aripiprazole and Risperidone
14. Risperidone and Quetiapine.
15. Which of the following best describes a major side effects associated with second generation antipsychotics.
16. Constipation
17. Sexual dysfunction
18. Weight gain
19. None of the above
20. Second generation antipsychotics are marked majorly with the following adverse effects **except**
21. Diabetes Mellitus
22. Weight gain
23. Hyperlipidemia
24. Neurologic side effects
25. Aripiprazole is an example of
26. Typical antipsychotics
27. Second generation antipsychotics
28. Third generation antipsychotics
29. None of the above
30. Mood disorders e.g depression should better be treated with
31. First generation antipsychotics
32. Atypical antipsychotics
33. Third generation antipsychotics
34. Tricyclic antidepressants
35. Schizophrenia is better treated with
36. Tricyclic antidepressants
37. Monoamine oxidase inhibitors
38. Typical and atypical antipsychotics
39. None of the above.
40. Antipsychotics are used in the treatment of the following except
41. Schizophrenia
42. Delirium
43. Delusional disorders
44. Parkinsonism.
45. The following except one should not be done in a someone just showing signs of psychosis.
46. Initiate therapy immediately
47. Isolate the patient
48. Withdraw from the person
49. None of the above.

| **Questions** | **YES** | **NO** |
| --- | --- | --- |
| 1. Antipsychotics can be sold as over the counter drugs (OTCs). |  |  |
| 1. Antipsychotics should be stopped once the patient show no more signs and symptoms. |  |  |
| 1. Atypical antipsychotics are more associated with neurologic side effects than typical antipsychotics. |  |  |
| 1. It is safer to place a patient with diabetes mellitus on atypical antipsychotics than on typical antipsychotics. |  |  |
| 1. One of the major side effects of typical antipsychotics is extrapyramidal symptoms. |  |  |
| 1. Butyrophenones e.g Haloperidol are classical examples of atypical antipsychotics. |  |  |
| 1. Sometimes, as a community pharmacist, it is better to initiate antipsychotic therapy in a patient especially with recurring signs. |  |  |
| 1. It is better to place patients on just one class of antipsychotics in order to enhance adherence. |  |  |
| 1. With a drug-naïve patient, it is better to initiate atypical antipsychotics than typical antipsychotics. |  |  |
| 1. Different classes of antipsychotics should be given to a patient that has been showing signs of psychosis for over a year. |  |  |

**SECTION C: THIS SECTION IS TO EVALUATE YOUR REASONS BEHIND DISPENSING ANTIPSYCHOTICS AS OVER-THE-COUNTER DRUGS.**

***Kindly tick “YES” or “NO” for questions 21-30 below***

|  | YES | NO |
| --- | --- | --- |
| 1. Sometimes, I dispense antipsychotics as over the counter drugs especially in an emergency. For instance, if a known patient runs out of his or her medication and is in urgent need. |  |  |
| 1. I do not request for prescription for antipsychotics when I’m dispensing to known patients. |  |  |
| 1. I believe every patient that walks into the pharmacy requesting for antipsychotics know what they want to use it for, so I dispense it when requested. |  |  |
| 1. I dispense the typical antipsychotics (e.g. Chlorpromazine, Haloperidol) as over-the-counter drugs but I ask for prescription for the atypical ones (Risperidone, Olanzepine). |  |  |
| 1. I readily dispense antipsychotics to older people because I believe they are matured enough to know what they want to buy. |  |  |
| 1. Sometimes, parents come to get antipsychotics for their children, and I readily dispense it especially when they tell me it’s for their children. |  |  |
| 1. I readily dispense antipsychotics when clients give good reports about the last brand they bought. |  |  |
| 1. I readily dispense antipsychotics to patients when they tell me they’ve been on it for long. |  |  |
| 1. I can switch to another class of antipsychotics when a patient gives complaints about the one he/she is currently on. |  |  |
| 1. I will rather dispense chlorpromazine to an obese patient rather than dispense clozapine because of the weight gain associated with clozapine. |  |  |
